# Food Waste and Consumer Behavior: A Bibliometric and Review Study and Future Research Directions

**DOI:** 10.3390/foods15020380

**Published:** 2026-01-21

**Authors:** Paula Karina Salume, Marcelo Werneck Barbosa, Marcelo de Rezende Pinto

**Affiliations:** 1 Department of Administrative and Accounting Sciences, Universidade Federal de São João del-Rei (UFSJ), Av. Visconde do Rio Preto, S/n, Bairro Colônia do Bengo, São João del-Rei 36301-360, Minas Gerais, Brazil; paulasalume@ufsj.edu.br; 2 Department of Agricultural Economics, Pontifícia Universidade Católica de Chile, Av. Vicuña Mackenna 4860, Macul, Santiago 7820436, Chile; 3 Institute of Economic and Management Sciences, Pontifícia Universidade Católica de Minas Gerais (PUC Minas), Av. Dom José Gaspar, 500, Coração Eucarístico, Belo Horizonte 30535-901, Minas Gerais, Brazil; marcrez@pucminas.br

**Keywords:** food waste, consumer behavior, bibliometric study, sustainable behavior

## Abstract

Despite an increasing number of studies on food waste, the research field on consumer behavior and food waste lacks a characterization of research specifically focused on how consumers behave regarding food waste. This study conducted a bibliometric analysis of literature on consumer behavior and food waste, characterizing the research community and identifying themes and emerging issues based on the Web of Science database. This study analyzed the evolution of the field over time and identified the most influential countries, authors, and sources, as well as the international collaboration networks in the area. We also highlighted the thematic trends in the research field of consumer behavior in food waste. In addition, we identified the methodological and contextual gaps cited in the literature in order to provide a future research agenda. This study concludes that the relationship between food waste and consumer behavior has become a pivotal axis of contemporary debates on sustainability, encompassing psychosocial determinants, household routines, and systemic and contextual factors that shape everyday food practices. Our findings also suggest a lack of integration between policies, interventions, and knowledge about FW determinants, calling for future studies to assess the effectiveness of interventions in altering consumer FW behavior. The findings of this study can contribute to the design of marketing campaigns and sustainable strategies for agri-food companies, as well as the development and improvement of public policies in this area.

## 1. Introduction

Food waste (FW) constitutes a critical global problem, with significant implications for consumers, environmental sustainability, and supply chain dynamics [1,2,3,4]. FW encompasses any edible material—whether raw, processed, or cooked—that is discarded, anticipated to be discarded, or otherwise excluded from human consumption despite being produced for that purpose [5,6]. In distinction, food loss occurs at earlier stages of the supply chain, including production, post-harvest handling, and industrial processing [5]. This phenomenon constitutes a critical environmental, social, and ethical challenge [7], with profound implications for sustainability [8]. Its impacts extend across ecological and societal dimensions [9], generating inequities by discarding food while significant portions of the population face hunger. The Food and Agriculture Organization (FAO) estimates that 33% of all food produced globally is wasted at some point along the supply chain [10].

Moreover, FW exacerbates environmental degradation, as overproduction and excessive consumption intensify pressures on natural resources [11]. From a social perspective, wasted food represents a missed opportunity to mitigate hunger and malnutrition, underscoring the centrality of food availability as a global concern [12]. Its relevance is unquestionable, representing a central challenge within the framework of the United Nations Sustainable Development Goals (SDGs), particularly target 12.3, which aims to reduce per capita FW by 50% by 2030 [9,13]. The 2030 Agenda emphasizes the importance of minimizing waste throughout all stages of the food supply chain, which also contributes to achieving Goals 2 (zero hunger), 13 (climate action), and 15 (life on land) [14,15].

A substantial portion of FW occurs at the final stage of the chain, with 46% of FW generated at the consumption stage, and 69% originating in households, food services, or retail [16]. Hence, households, in particular, account for a disproportionately high share of FW compared to other stages of the supply chain [8,17]. At the consumption stage, waste is closely tied to purchasing behavior, making domestic FW particularly relevant for analysis and intervention. This focus offers significant opportunities to design strategies that effectively reduce waste [18]. Consumer habits are widely recognized as the primary drivers of FW [5]. Preventing surplus food from being discarded throughout the supply chain, especially at the consumption stage, requires a clear understanding of behaviors that can mitigate final disposal [12]. The negative impacts of the food industry on resource scarcity and environmental degradation are linked mainly to consumer practices [19]. Waste generated at the household level has severe societal consequences, as both environmental costs and economic value accumulate along the chain [20]. Since consumers generate waste in diverse settings—including households, restaurants, and other venues—examining their knowledge, attitudes, and behaviors is a critical area of study [9].

In high-income economies, such as those in Europe and North America, FW is predominantly caused by distribution and household-level consumption [21,22,23], where per capita consumption is estimated to result in the discard of between 95 and 115 kg of food annually—a volume that exceeds the waste generated by the food service industry [24]. Beyond representing a financial cost—families are estimated to spend around five euros per week on food that will be discarded [1]—the disposal of uneaten food results in higher greenhouse gas emissions, greater resource use, and biodiversity alteration than necessary [24].

Consumers are significant contributors to FW, with household FW often attributed to carelessness and negligence [25,26]. Key behavioral drivers include poor planning, preparation of excessive portions, and misinterpretation of expiration terms such as “use by” and “best before.” FW predominantly occurs after preparation due to this misunderstanding of labels [21,27]. Moreover, household FW behavior is linked to the household food provisioning process, which involves interrelated decisions on planning, purchasing, storage, and preparation [21,28].

Previous research has revealed that over-purchasing and the desire to be a “good provider” by preparing excessive amounts of food often result in waste [21,24]. Conversely, meal planning routines and leftover reuse practices are important predictors of lower levels of FW [29,30]. At the motivational level, consumers are primarily driven by financial interest (seeking to save money) and a desire to avoid waste [31]. Beyond the household context, the hospitality sector is identified as fundamental to sustainable FW management, with practical strategies involving management, staff training, and influencing consumer behavior [32,33,34].

The literature on the subject has been fundamentally based on psychosocial models, with the Theory of Planned Behavior (TPB), proposed by Ajzen [35], serving as the central framework [1,9,21,33,36]. TPB establishes that behavioral intention is the primary antecedent of behavior, determined by attitudes, subjective norms, and perceived behavioral control. However, applying TPB in the context of FW often requires extensions [21,24]. For example, the model has been extended to include contextual factors such as household food management routines and practices, as waste is a behavior that is often not fully under intentional control [21,24,29]. Perceived behavioral control, which represents the perceived ease or difficulty of engaging in a behavior [35], can directly determine FW through food-related routines, not only through intentions [21,24,29].

Addressing household FW requires greater attention to innovative intervention strategies [7]. Recognizing the factors that shape consumer behavior—including attitudes, motivations for waste, and waste quantities—is essential for developing effective prevention strategies [37]. At the international level, the issue of FW has assumed growing importance, reflected in the significant rise in scientific output in recent years [18]. Literature on household FW has expanded rapidly [15], with numerous studies published since the onset of the COVID-19 pandemic, particularly during lockdown periods across different countries [14,38]. Nevertheless, research that explicitly prioritizes consumer behavior in tackling FW remains limited [9], and systematic, chronological, and synthesizing studies tracing the evolution of this field are still lacking [39]. Bibliometric analysis offers a valuable approach to identifying influential studies, tracking research trends, and methodologies employed in FW research [40].

Previous studies have investigated FW behavior through bibliometric studies. Gál et al. [1] analyzed recent FW literature using Web of Science (WoS) and Scopus, identifying research gaps in sustainable FW management. Zhang et al. [41] examined research activities and trends from 1991 to 2015 through bibliometric analysis. Widayat et al. [9] conducted a systematic review of FW behavior across management, business, economics, and social sciences, identifying clusters related to individual practices, regulatory frameworks, waste characteristics, sources, and production processes [5]. Moraes et al. [42] reviewed empirical studies on prevention and minimization methods, while Batool et al. [43] focused on the environmental consequences of FW using life cycle assessment techniques. Wang et al. [44] proposed a conceptual framework for evaluating the environmental impact of reducing agri-food waste, based on a bibliographic analysis. Table 1 synthesizes the main characteristics of these closely related review and bibliometric studies on FW.

Table 1 shows that several review studies have been conducted on FW; however, they have different focuses, including FW management, agri-food waste management, FW drivers, FW and sustainability, and FW interventions, among others. Nonetheless, these studies have not covered the intersection between FW and consumer behavior. The only exception is the work of Widayat et al. [9], which has been characterized as an SLR performed solely on the Scopus database. Our study extends these related reviews by conducting a bibliometric analysis of the literature on FW and consumer behavior in high-reputation journals indexed in the WoS database.

In light of these gaps, the present study conducted a bibliometric analysis of the literature on consumer behavior and FW, identifying patterns, collaborations, and emerging issues based on the WoS database. Bibliometrics becomes important as a tool capable of filtering documents, providing insights into trends and impact, mapping the intellectual structure of the field, and proposing a future agenda aligned with real needs for theoretical and applied advancement [1,46].

This study seeks to address the following questions: RQ1: How has research evolved? RQ2: Which countries, authors, and sources are most influential and productive, and how are international collaboration networks configured in this domain? RQ3: What are the publication patterns and dominant thematic trends (including Motor, Basic, and Emerging Themes) that structure the research field on consumer behavior in FW? Finally, RQ4: Which methodological and contextual gaps cited in the literature provide a future research agenda for a more integrated study of consumer FW?

This study makes a significant contribution to the field by mapping the thematic evolution, while also presenting theoretical and methodological advances that inform researchers about areas of greatest impact and those that are emerging. Additionally, it facilitates the mapping of authors, networks, and schools of thought that have driven the most influential debates, providing support to strengthen international collaborations and broaden the epistemological diversity of the field. On the practical dimension, the results, by identifying key factors, can assist policymakers and stakeholders in adopting data-driven strategies to reduce FW, such as educational programs or awareness campaigns. The findings may also guide more evidence-based public policies and market strategies, highlighting which behavioral factors are most sensitive to intervention and which contextual dimensions remain underexplored. Thus, this study aims not only to highlight the progress achieved but also to establish the foundations for more targeted and impactful efforts to address this critical challenge. It should also be emphasized that this study not only organizes existing production but also points to pathways for developing more robust models, more precise methodologies, and multi-scalar approaches capable of enhancing the understanding and mitigation of FW in consumers’ everyday lives.

## 2. Materials and Methods

Bibliometric analysis is a systematic and quantitative method that applies statistical tools to large samples of publications in order to identify research trends and structural characteristics within a field [1]. It examines publication patterns, keywords, citations, and co-authorship networks to provide a comprehensive overview of the research landscape and highlight influential authors and themes [47]. More broadly, bibliometric analysis measures the output of individuals, institutions, and countries, maps collaboration networks, and traces the development of multidisciplinary fields of science and technology [48].

The research process used in this study was systematized and adapted from Tranfield et al. [49], do Prado et al. [50], and Costa et al. [51], and comprised five main stages, detailed in the subsections below: Planning, Data Collection, Data Selection and Organization, Data Analysis, and Reporting.

In the planning phase, the scope of the study was defined as an in-depth analysis of scientific articles focused on consumer behavior in relation to FW, based on WoS articles. WoS has been chosen since it covers several high-ranking and reputable journals as well as high-quality peer-reviewed articles [52]. This database has been used in previous bibliometric studies [53,54]. Data collection was carried out in October 2025, using the WoS database. The search string used to ensure the relevance of documents was: TS = (“consumer behavio*” OR “consumer attitude*” OR “consumer decision*” OR “consumer choice*” OR “consumer awareness”) AND TI = (“food waste” OR “food loss” OR “food discard*” OR “food surplus” OR “food wastage” OR “wasted food”), where

• TS (Topic) searches terms in the title, abstract, and keywords fields.
• TI (Title) searches terms only in the title.
• OR and AND are Boolean operators that combine search terms.
• * is a wildcard character that allows inclusion of word variations (e.g., “behavior” and “behaviors”).

The use of these criteria for the search was based on previous research that recommended limiting the search terms to the article titles [55] and including terms in quotation marks to achieve more precision and accuracy in the retrieved records [56].

Applying this search string resulted in 336 documents. To refine the sample, the option “Show Final Publication Year” was selected, which reduced the records. Additionally, filters were applied: (i) documents only of type “Article,” reducing the sample to 275; and (ii) articles written in English, resulting in a final base of 274 records. No restrictions on publication date or scientific field were applied at this initial stage, aiming for the broadest possible coverage.

In the Data Selection and Organization stage, the 274 initially identified articles were submitted to a selection process by specialists to ensure their relevance and adherence to the study’s scope. The evaluation was conducted through titles and abstracts, with each article reviewed by at least two independent specialists, who analyzed the title and abstract to verify direct pertinence to consumer behavior and FW.

The following inclusion criteria were defined: articles explicitly addressing the theme of “food waste” or “food loss,” and articles whose study context focused on “consumer behavior in relation to food waste.” The exclusion criteria included articles addressing FW in other links of the production chain (e.g., agricultural production, industrial processing, retail) without a focus on consumer behavior; articles not directly related to FW (e.g., general food security, nutrition without focus on losses, carbon footprints, recycling); and duplicates. Two experts analyzed the set of papers to apply the inclusion and exclusion criteria, considering the title, abstract, and keywords to determine whether each article was aligned with the study’s scope. Discrepancies between the two experts were discussed and addressed. At the end of this process, a final sample of 229 articles was included in the bibliometric analysis. The complete bibliographic records of these articles (authors, titles, abstracts, keywords, citations, publication information) were downloaded from WoS in a compatible format.

The data analysis phase was conducted primarily using the Bibliometrix package and the Biblioshiny application within the R and RStudio (2025.09.1 Build 401) environment, with support from Microsoft Office Excel for visualizing and organizing raw data, as well as preparing graphs and tables. In this study, Biblioshiny (version 5.2.1) was employed to generate the production map, collaboration networks, and thematic map. This approach facilitated the identification of patterns, the visualization of complex relationships, and the understanding of the behavior of studies in the field.

The bibliometric analyses conducted covered the following categories:

• Overview of the database and research evolution: analysis of the temporal distribution of publications and citations, focusing on identifying periods of growth and stagnation, and calculation of indicators such as the h-index to assess the aggregate impact of the database.
• Production and geographic collaboration map: visualization of scientific production by country and the intensity of international collaboration, identifying central and peripheral research hubs.
• Leading authors: identification of the most productive authors in terms of the number of publications, their affiliations, and period of activity.
• Collaboration networks among authors: analysis of co-authorship relations to identify research communities, collaboration hubs, and knowledge flows within the field.
• Thematic map: generation of a thematic map to visualize the most relevant topics and their development in the field, categorizing them into Motor Themes, Niche Themes, Basic Themes, and Emerging or Declining Themes.
• Most cited articles: compilation and analysis of the most impactful articles, based on total citations and average annual citations, with an in-depth discussion of their main findings, methodologies, and contributions.

In the reporting phase, the results of the analyses were presented clearly and systematically, using tables, figures, and text. The organization of results followed the categories of analysis described above, enabling a comprehensive understanding of the research landscape on FW in food services. This report provides a solid foundation for future discussions and the identification of opportunities for research and interventions.

## 3. Results

### 3.1. Overview of the Database and Research Evolution

**Figure 1:**
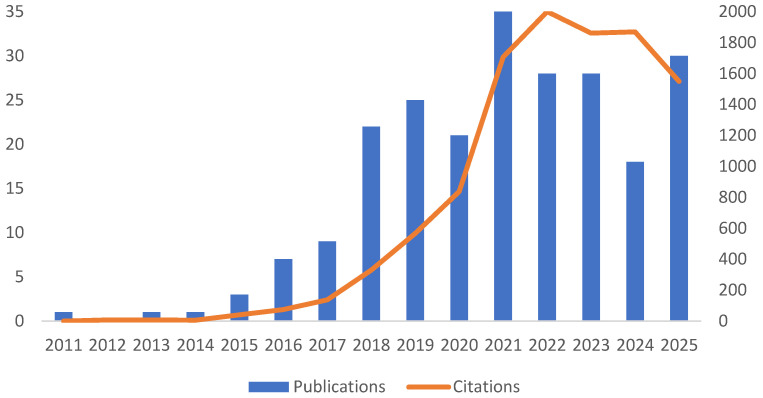
Growth of publications and citations. Source: Bibliometrix, based on WoS data (2025).

The analyzed set of papers comprises 229 articles on consumer behavior in relation to FW. Figure 1 presents the growth of publications and citations and its impact over a fifteen-year period, from 2011 to 2025, addressing RQ1. The information is represented by annual publications (blue bars) and the total citations received each year (orange line). Figure 1 depicts the growth and recognition of this research activity.

The analysis of publications reveals that few articles have been published between 2011 and 2014, with academic output limited to one or two publications per year. This initial scenario illustrates a still-emerging field that has received limited attention from the scientific community. However, from 2015 onwards, a significant inflection is observed, with the number of publications rising to three and initiating a trajectory of steady growth that reached 25 articles in 2019. The first major peak occurred in 2021, with 35 publications, followed by a period of relative stabilization, with an average of 28 annual publications between 2022 and 2023. Following a reduction to 18 publications in 2024, projections for 2025 indicate 30 or more articles, based on partial data available (search conducted in October 2025), which suggests the maintenance of a high level of scientific productivity on the topic.

The sharp growth from 2015, with important milestones in 2021 and a projected recovery in 2025, points to multifaceted drivers that have propelled the development of this research area. Among the most significant elements is the global recognition of FW as a critical sustainability challenge, particularly following the adoption of the United Nations SDGs in 2015, with a particular emphasis on SDG 12.3, which addresses FW. This political-institutional milestone has catalyzed greater research funding and stimulated broader engagement from the scientific community. In addition, methodological advances in consumer behavior analysis, including new techniques for data collection and processing, have certainly contributed to expanding and deepening investigations in the field.

Citation data present a distinct yet complementary dynamic. During the initial period (2011–2015), citations remained at insignificant levels (0–40 citations/year), reflecting both the reduced volume of publications and the time required for the academic community to disseminate and assimilate the work. From 2016 onwards, an upward curve is observed, intensifying especially between 2018 and 2021, when citations jumped from 330 to 1706. The peak occurred in 2022, with 2000 citations, followed by slight oscillations in subsequent years (1861 in 2023 and 1869 in 2024), and a projected number of 1549 citations for 2025, with the year still in progress.

The comparative analysis between publications and citations reveals a positive correlation but is marked by a characteristic temporal lag. The accelerated growth in production from 2015 precedes by about three years the significant increase in citations, which only gained momentum in 2018. This phenomenon clearly illustrates the time required for scientific work to achieve its full impact. The leap in citations between 2020 (837) and 2021 (1706), when they nearly doubled in a single year, highlights the growing recognition of the topic’s relevance. It is particularly significant that, in 2022, citations reached their absolute peak (2000) despite a smaller number of publications compared to 2021, indicating that articles published in previous years, especially during the productive peak of 2021, continued to exert a strong influence. The projected decline in citations for 2025, despite the expected increase in publications, may reflect both the time needed for more recent work to accumulate citations and a possible temporary saturation in the field. The implications of these findings are positive, demonstrating that researchers have not only significantly expanded academic production on a socially relevant topic—consumer behavior toward FW—but also generated a growing scientific impact in their area of activity.

The assessment of the field’s maturity and impact can be deepened through analysis of the h-index and average citations per article. The h-index of 54 indicates that 54 studies in this set received at least 54 citations each, representing a solid core of influential production. These studies collectively account for 7831 citations, representing approximately 71% of the total citations during the period (7831/10,989). Complementarily, the high total average number of citations per article (48) reinforces the qualitative recognition of scientific output in the area.

These indicators consolidate the perception that the study of consumer behavior in relation to FW has moved beyond the emerging phase to establish itself as a mature and recognized field. The existence of a significant corpus of widely cited works that serve as references for new research characterizes an area with robust theoretical and empirical foundations. This academic maturity has relevant practical implications, as it provides a consistent scientific basis for the formulation of public policies and sectoral strategies aimed at reducing FW, with the potential to impact both environmental sustainability and global food security positively.

### 3.2. Global Production and Collaboration Map

**Figure 2:**
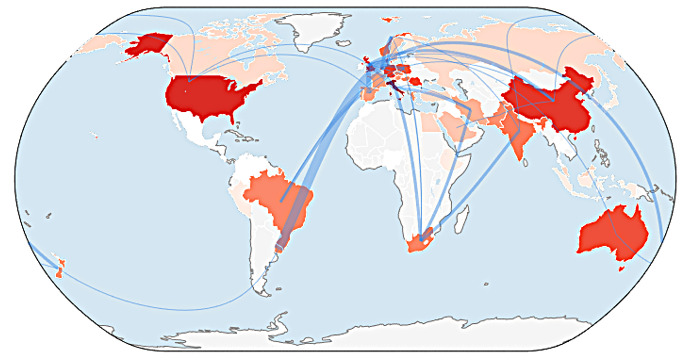
Global production and collaboration map. Source: Bibliometrix, based on WoS data (2025).

Figure 2 illustrates the global production and collaboration map on consumer behavior and FW, addressing RQ2. The colors applied to countries represent the volume of scientific production, with darker shades indicating higher output. A set of lines represents collaboration connections between nations, with thicker lines between two countries indicating more intense collaboration, reflecting greater integration into international scientific networks. The contrast between areas of high and low chromatic density allows identification of research poles and peripheries on the topic. The network of lines reveals the flows of knowledge co-production and the degree of centrality of certain countries in terms of collaboration.

The distribution of colors on the map, associated with the volume of scientific production by intensity, reinforces the asymmetry of the network. Italy stands out as the country with the highest scientific output, followed by major players such as the United States of America (USA), Russia, China, and Denmark, as well as other European centers, including the United Kingdom and Germany. Italy’s prominence can be attributed to a combination of factors, including a historically consolidated university ecosystem, the presence of robust research organizations, intense participation in European consortia, and access to large scientific infrastructures. In the USA, the scale of public and private investment, the dynamism of the university sector, and strong industry interfaces may underpin its elevated level of production. Russia maintains a tradition of excellence in fundamental areas, such as physics and mathematics. China has significantly expanded its investment in Research and Development over the past few decades. Denmark, though smaller in population, achieves high per capita productivity and a substantial impact in areas such as health and biotechnology. Thus, funding intensity, institutional critical mass, advanced infrastructure, and national specializations help explain the higher positions in the ranking of scientific production on consumer behavior and FW.

The connection lines suggest collaboration patterns at two levels: one represented by a dense intraregional circuit and the other by intercontinental axes. In Europe, an intricate network connects Italy to its major neighbors, including Germany, the United Kingdom, and other EU countries, reflecting the possible existence of multinational consortia and collaborative funding programs. From a transcontinental perspective, frequent connections emerge between European centers and the USA, which may stem from large projects, co-authorships in high-impact journals, and consolidated thematic networks. Collaboration strands also connect these poles to countries in other regions, such as Brazil in South America, South Africa in Africa, and Oceania nations such as Australia and New Zealand. China and Russia appear as hubs with distributed connections, linking to both European partners and countries in Asia and other continents, revealing a diverse global reach likely resulting from disciplinary and strategic affinities.

The map suggests that large volumes of production, combined with extensive collaboration networks, function as engines of scientific advancement [57], the diffusion of practices, and the acceleration of research on consumer behavior and FW. However, concentration in a few poles may exacerbate asymmetries, generating dependence on external resources and agendas in countries with lower installed capacity. Therefore, by highlighting where production is concentrated and how collaboration occurs, the map provides an important diagnostic tool for adjusting scientific policies, consolidating existing partnerships, and creating new connections that can accelerate research and address global challenges, particularly those as relevant as FW.

### 3.3. Main Authors

#### 3.3.1. Top Publishing Authors

To characterize the authorship structure of the topic under analysis—consumer behavior and FW—we identified the researchers with the highest volume of publications in the set of articles considered. Identifying these authors enables us to identify intellectual references, collaboration centers, and potential schools of thought, in addition to guiding priority readings and identifying potential partnerships. Table 2 presents the top ten publishing authors. Additionally, it displays the current affiliation of the researchers, the publication years of the articles, and the percentage of publications in relation to the set of papers investigated (229 articles).

Regarding institutional affiliations, there is geographic diversity among the ten leading authors, involving institutions in Denmark, Uruguay, Italy, the United Kingdom, Hungary, and Australia. However, two institutional poles stand out: Aarhus University, to which Jessica Aschemann-Witzel and Gaston Ares are affiliated, and the University of Bologna, which brings together Matteo Vittuari, Luca Falasconi, Andrea Segre, and Marco Setti, who have co-authored works. The presence of multiple authors from the same institution suggests a mature research environment, with infrastructure and networks capable of sustaining collaborative agendas. This context tends to further increase the likelihood of co-authorship through data sharing and integrated projects, thereby boosting both individual productivity and collective impact. This institutional maturity is evident in specialized infrastructures and internal collaborative networks that support long-term research agendas, as demonstrated by studies examining the relationship between intra-organizational network density and scientific performance [58].

The ability to maintain cohesive groups of researchers within the same institution not only facilitates the systematic sharing of data and resources but also promotes the formation of a more robust bond between collaborators, a mechanism identified as a positive mediator between centrality in collaborative networks and scientific production [59]. This internal synergy fosters an ecosystem that promotes the development of integrated projects, which transcend individual approaches, thereby enhancing both academic productivity and the collective impact of the research generated. Studies on hyper-authorship demonstrate that, although works with an excessive number of authors may distort network metrics, institutionally anchored collaboration tends to produce more substantive and strategically aligned co-authorships [60]. This fact is particularly relevant in contexts where shared project experience strengthens collaborative ties, creating networks of trust and reciprocity that transcend individual projects [59]. Thus, the identified institutional hubs not only reflect installed capacity but also act as catalysts for synergies that amplify scientific returns in both quantitative (in terms of the number of publications) and qualitative (in terms of citations and impact) terms.

#### 3.3.2. Authors with Greatest Impact

For the identification of the most impactful authors, bibliometric metrics commonly employed were considered: h-index, g-index, and m-index, which, although related, capture distinct dimensions of scientific performance. Previous research has reported that using the h-index as the sole indicator for assessing researchers’ careers is not recommended. Thus, the h-index should be complemented with other indicators [61].

The h-index is a measure that assesses both productivity and research impact. The h-index was introduced by Hirsch [61] and is defined as follows: “A scientist has index h if h of his or her Np papers have at least h citations each and the other (Np-h) papers have fewer than h citations each.” The g-index [62] measures a researcher’s impact based on their most cited publications. It considers a set of articles ranked in descending order by citation count, thereby increasing sensitivity to “anchor articles.” A g-index of 10 means that the 10 most cited articles of an author together have at least 102 (100) citations. The m-index is an indicator calculated by dividing a researcher’s h-index by the number of years of their career, normalizing the h-index, and enabling comparisons between early-career researchers and those with longer academic experience [63]. Table 3 presents the most five impactful researchers, considering the h-index, g-index, and m-index, as well as the initial year of publication on the topic of consumer behavior and FW.

The integrated analysis of the five most impactful authors reveals a cohesive body of research around the thematic axis of FW reduction from the perspective of consumer behavior. This work articulates, in complementary fashion, psychosocial constructs (personal norms, religious values, family socialization), perceptions and decisions in purchasing/consumption (perceived quality, acceptance of suboptimal foods, price and convenience orientation), and contextual and structural dynamics (retail environment, exogenous shocks such as COVID-19, household income and resource constraints). Three interconnected macro-themes emerge: (1) Acceptance and value of suboptimal foods as a tool for waste prevention, (2) Formation and activation of norms and motivations to avoid waste, with emphasis on cultural and religious mediations, and (3) Consumer decision-making in retail and households, influenced by lifestyle segmentation, communication, and contextual conditions of uncertainty.

In the first macro-theme, Aschemann-Witzel plays a central role by proposing a functional definition of upcycled food, distinguishing alternative uses and innovative uses as complementary pathways to add value and reduce losses [64]. In recurring collaboration with Ares and Gimenez, she demonstrates that communication oriented towards waste reduction and price repositioning increases acceptance of suboptimal products, with variations by category and sociodemographic profile. Studies with European and Latin American consumers converge in showing that acceptance depends on perceived quality dimensions and trade-offs between price, convenience, and value, and that lifestyle segments differ in their propensity to waste and to choose imperfect items [65,66,67]. These results align with experimental evidence on fruits, where external defects and internal browning modulate visual attention and disposal, suggesting that interventions should balance messaging by category and severity of defects to expand consumption of products still fit for ingestion.

The second macro-theme is consolidated by Filimonau et al., who demonstrate in multiple contexts (Turkey, Poland) that religious values, filial piety, and family socialization operate as antecedents and mediators of personal norms to avoid waste, with effects modulated by social distance [68,69]. In food service environments, such values activate compassion and reinforce normative injunctions when interactions occur within close social circles (family, friends), pointing to pathways for campaigns that mobilize moral emotions and intergenerational references [70]. Extending the analysis to retail and operations management (e.g., cafeterias), institutional and governance barriers (such as legislation and internal resources) emerge that limit preventive strategies, reinforcing the need for coordinated policies that align business practices with consumer behavioral change [71].

The third macro-theme organizes decisions in retail and households under real purchase and use conditions [72,73]. In an extended collaboration, Aschemann-Witzel demonstrates that consumers evaluate the feasibility of future consumption already in-store, weighing factors such as packaging unit, expiration date, perceived quality, and household storage/planning routines [74]. Furthermore, price-oriented profiles report lower waste, whereas convenience-oriented profiles tend to increase it. Gimenez et al. [75] in qualitative and survey studies in emerging countries, detail that categories such as fresh produce and leftovers concentrate disposal due to suboptimality and prolonged storage, and that men and young people emerge as priority targets for communication. This block connects to a structural dimension led by Vittuari and collaborators, who show, through structural equation modeling, that uncertainty induced by COVID-19 reconfigured household resources and routines, reducing self-reported waste during quarantine; and, through economic analyses and agent-based models, that income, social interactions, and cognitive openness modulate private waste dynamics and opinion-behavior equilibria over time [76].

By combining the macro-themes extracted from the publications of the five most impactful authors, it is possible to observe that high g-index values among leading authors are associated with “anchor articles” that define concepts (e.g., upcycling), decision frameworks in retail, and psychosocial mechanisms (moral norms, socialization). Meanwhile, the h-index sustains the consistency of empirical applications across multiple countries and methods (interviews, experiments, surveys, among others). The m-index distinguishes rhythms of impact consolidation: more accelerated when conceptual definition, experimental validation, and recommendations for policy and retail are articulated (Aschemann-Witzel; Ares; Gimenez), and equally robust when macro shocks (COVID-19) and socioeconomic structures (income) are integrated into behavioral mechanisms (Vittuari), or when normative and cultural dimensions are deepened in out-of-home consumption contexts (Filimonau). Thematic coherence, methodological complementarity, and co-authorship explain the predominance of these five authors in the sample.

### 3.4. Collaboration Networks Among Authors

**Figure 3:**
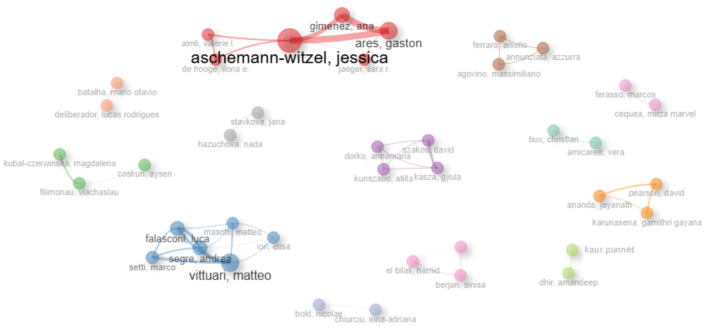
Authors’ collaboration networks. Source: Bibliometrix, based on WoS data (2025).

Figure 3 presents the authors’ collaboration networks. It synthesizes, in a single visualization, the relational structure of a research field. The nodes correspond to individual authors; their size reflects prominence in the network (associated with productivity and centrality). The lines indicate co-authorship relations, with their thickness encoding the strength of these collaborations (frequency and intensity). Coloring differentiates communities detected through clustering, highlighting groups that collaborate more intensively among themselves than with the rest of the network.

The co-authorship collaboration network reveals a modular structure, with communities clearly delineated by color and arranged asymmetrically across the plane. At the top left, a red community stands out, densely connected and anchored by a larger node associated with Aschemann-Witzel, around whom Almli, Gimenez, and Ares gravitate. At the bottom left, a light-blue community of greater spatial extension emerges, in which Vittuari and Falasconi are prominent, together with Italian collaborators, reflecting an agenda focused on modeling, income, and exogenous shocks. The internal configuration suggests recurrent co-production and a high degree of intracluster interconnection.

Around these two main communities, smaller, chromatically distinct groups emerge, denoting specialized subcommunities with cohesive internal ties and weaker external connections. Within this set, a medium-blue subgroup associated with Filimonau stands out. Filimonau, listed among the most impactful authors, collaborates with characteristic co-authors such as Kubal-Czerwinska and Coskun in research related to personal norms, religious values, and out-of-home food environments.

Intercommunity relations are visibly less numerous and, when present, tend to be thinner, indicating weaker ties between communities. In addition, weak collaboration networks pose a challenge for the development of the field [77,78]. The most central authors function as potential bridges for knowledge diffusion: Aschemann-Witzel’s community connects acceptance/consumption agendas to other fronts. In contrast, Vittuari’s community articulates structural and contextual approaches. Taken together, the network highlights a pattern of thematic specialization with selective integration, in which a few hubs—notably Aschemann-Witzel and Vittuari, with the co-participation of Ares, Gimenez, and Filimonau—sustain global connectivity and foster the circulation of results across subfields. This arrangement, combined with the g-index for anchor articles, the h-index for consistency, and the m-index for consolidation pace, explains the prominence of these five authors [58,59].

### 3.5. Thematic Mapping

**Figure 4:**
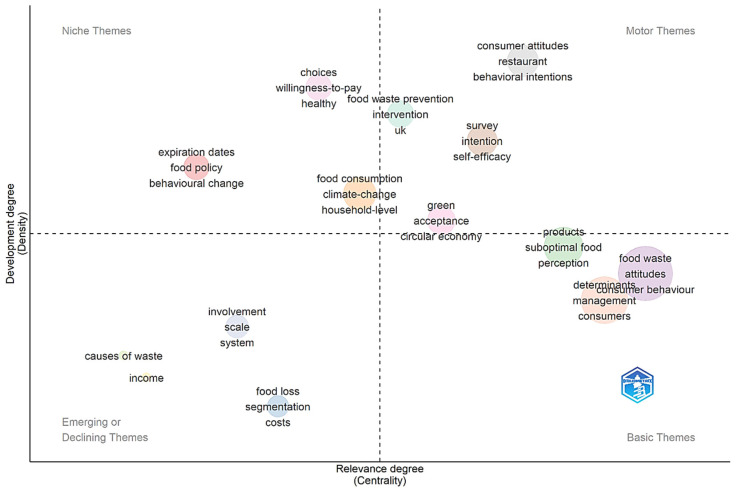
Thematic map. Source: Bibliometrix, base on WoS data (2025).

The analysis of the thematic map reveals a research structure that, although robust in its psychosocial foundations, exhibits critical gaps in the integration of solutions and in expansion toward multilevel and socioeconomic contexts. The thematic map, presented in Figure 4, addresses RQ3 and provides an overview of research on consumer behavior and FW, using two main dimensions: degree of relevance or centrality (X-axis), which measures the importance and interconnection of a theme with others, and degree of development or density (Y-axis), which indicates the maturity and depth of internal research on a specific theme. The combination of these metrics defines four quadrants: (1) Motor Themes (high relevance and high development), (2) Niche Themes (low relevance, high development), (3) Basic Themes (high relevance, low development), and (4) Emerging or Declining Themes (low relevance, low development).

The core of the field lies in the Basic Themes and Motor Themes, which occupy a central position of high importance. Basic Themes, including FW attitudes, consumer behavior, and determinants, form the conceptual foundation of research. Although essential, their low density suggests that understanding how these determinants interact with factors such as products and suboptimal food still lacks methodological depth to mature into a consolidated field of study. The centrality of Basic Themes reveals a fundamental theoretical tension in the field. While these concepts form the conceptual foundation, their low density mirrors the methodological debate between psychological reductionism and systemic approaches. As argued by Evans [79] and Shove [80], the predominance of individual psychological determinants reflects a theoretical tradition that privileges micro-level explanations over macro-level explanations, thereby limiting the understanding of how contextual factors (such as products and suboptimal food) interact with attitudes. This methodological gap impedes field maturation because robust theories require not only variable identification but models that capture complex interactions between multiple levels of analysis.

Motor Themes, in turn, demonstrate the maturity and strength of the area. The high density and relevance of terms such as behavioral intentions, intention, and self-efficacy confirm the dominance of models, including the TPB, in explaining consumer behavior. This concentration reflects an ongoing theoretical debate between models of deliberate rationality [35] and practice theories [81] that emphasize unconscious routines. However, the central positioning of the term survey in this methodological quadrant highlights a relevant limitation of the field—namely, its strong dependence on self-reporting, which induces social desirability bias and compromises data accuracy, reinforcing the need to migrate toward more objective measurement methods. The presence of restaurants as a Motor Theme, in turn, signals an important theoretical expansion—the transition from exclusive focus on households to institutional contexts, aligning with Principato et al. [82] call for theories that consider different consumption logics in distinct food environments.

The main structural gap lies in the relationship between Niche Themes and Basic Themes. Niche Themes, such as food policy, intervention, and behavioral change, exhibit high density, meaning they are well-developed subfields. However, their low centrality indicates that the solutions and policies developed are not yet intrinsically linked to the core behavioral determinants. This disconnection suggests that knowledge about “what to do” (policies and interventions) has not been fully integrated with knowledge about “why people waste” (determinants), requiring future research that models the effectiveness of interventions in directly altering consumer attitudes and routines. The structural disconnection between Niche Themes and Basic Themes materializes a critical theoretical gap: the division between descriptive science and prescriptive science. As theorized by Michie et al. [83], effective policies require theoretical alignment between behavioral determinants and intervention strategies. The low centrality of policy themes reveals that the field has not yet resolved the theoretical debate on how micro determinants (attitudes) connect to macro changes (policies). This separation mirrors Shove [80]’s critique of the “ABC ethics” (Attitude-Behavior-Choice), which prioritizes individual changes over systemic transformations. To overcome this gap, it is necessary to develop mediation theories that, as proposed by Vermeir et al. [84], explain causal mechanisms through which policies alter established routines.

Finally, the quadrant of Emerging or Declining Themes points to the most neglected areas. The low density and centrality of terms such as system, scale, and income reveal that the field has focused excessively on the individual level, overlooking multilevel complexity and systemic interactions. The marginalization of Emerging or Declining Themes exposes a profound theoretical limitation: the methodological anthropocentrism dominating the field. The neglect of system and scale reflects the persistence of theoretical models, as criticized by Spaargaren [85], that treat waste as an individual behavioral problem, ignoring social-ecological systems theories [86] that emphasize multi-level interactions. The underexploration of income reveals a geographical theoretical bias: the uncritical application of Western psychological models (such as TPB) to contexts where economic theories of scarcity would be more explanatory.

### 3.6. Most Cited Articles

This section presents a compilation of the ten most influential and widely cited articles in the literature on consumer behavior and FW, identified based on the average number of citations per year within the investigated database. Citation metrics serve as indicators of the influence, dissemination, and scientific impact of publications within the academic community. Table 4 presents the titles of the ten most cited articles, their authors, year of publication, average citations per year, and total citations.

The ten articles analyzed in this investigation, published between 2013 and 2023, accumulated 3087 citations, evidencing their significant impact on contemporary scientific literature and their centrality in the field.

The most cited work, “Determinants of Consumer Food Waste Behaviour: Two Routes to Food Waste” [21], constitutes a theoretical turning point in the field. Conducted with 1062 Danish consumers, this article proposed an alternative model by identifying two distinct routes to FW behavior: the intentional route, grounded in the TPB, and the routinized route, centered on everyday habits and food practices. The main findings revealed that, contrary to initial expectations in the literature, perceived behavioral control and routines related to planning, purchasing, and leftover reuse are the primary drivers of FW—far more than purely intentional factors. The study demonstrated that these routines are intrinsically linked to consumers’ perceived ability to manage household food-related activities.

In second place, the study “Sorting Out Food Waste Behaviour: A Survey on the Motivators and Barriers of Self-Reported Amounts of Food Waste in Households” [24] expanded understanding through a survey of Swiss consumers, examining specific motivators and barriers to FW across eleven distinct food categories. This work stands out for its emphasis on psychosocial constructs beyond traditional TPB, particularly by identifying the “good provider identity” as a critical, and often overlooked, factor in the previous literature. The desire to provide sufficient food for family and guests often paradoxically leads to greater waste. The main findings showed that perceived behavioral control and the good provider identity emerge as central motivators rather than barriers. Conversely, the study revealed that different predictors are significant for distinct food categories: price awareness proved important for expensive products such as meat and fish, while environmental concerns played only a secondary role in motivating waste reduction.

The progressive understanding that FW is not merely a matter of individual psychology or lack of awareness but is rooted in complex practices shaped by structural contexts has led researchers to explore natural events as opportunities for investigation. In third place, the article “COVID-19 Virus Outbreak Lockdown: What Impacts on Household Food Wastage?” [87], published during the pandemic, examined a natural experiment—the impacts of the pandemic lockdown on household FW behavior in Tunisia. Contrary to initial expectations, the study found a significant reduction in FW during lockdown, attributed to increased awareness of the importance of food, more careful planning of purchases driven by the need to limit outings, and a greater intention to reuse leftovers.

The importance of planning and shopping routines, identified in earlier works, was further emphasized in a study titled “Avoiding Food Waste by Romanian Consumers: The Importance of Planning and Shopping Routines.” [29]. Ranked fourth, this article, conducted with Romanian consumers, offered one of the first robust demonstrations that structured planning and shopping routines are predictors of FW when compared to purely psychological constructs. The central findings revealed that these routines are fundamental determinants of self-reported waste, themselves shaped by moral attitudes toward waste and perceived behavioral control, while feelings of guilt emerged as relevant mediators between moral attitudes and actual behavior. A unique contribution of this work was its demonstration in a post-socialist context, suggesting that the identified factors have cross-cultural validity.

While these early works consolidated theoretical frameworks for understanding waste in European household contexts, research began to explore the critical gap between awareness and actual behavior. In fifth place, the study “Wasted Food: U.S. Consumers’ Reported Awareness, Attitudes, and Behaviors” [31], investigated awareness, attitudes, and behaviors of U.S. consumers through a survey, documenting an important paradoxical finding: although 66% of respondents described themselves as making “much effort” to minimize FW, estimates of actual waste and reported behaviors suggested significant underreporting, reflecting social desirability bias. Among reported motivations to reduce waste, saving money was the leading factor, while environmental concerns ranked only tenth, revealing that financial motivations outweigh environmental ones.

Expanding the horizons of the field even further, recent researchers have begun to explore not only waste reduction but also the transformation of food residual streams into valuable consumable products. In sixth place, the article “Defining Upcycled Food: The Dual Role of Upcycling in Reducing Food Loss and Waste” [64] represents a turning point in the field. The article defines upcycled food as products created from ingredients deemed unsuitable for human consumption, transformed into nutritious and palatable foods through technological and culinary innovation. This concept offers a more economically viable alternative than simple behavioral reduction: instead of persuading consumers to waste less, it captures economic value from residual streams. The article demonstrates that upcycling operationalizes the principle of the circular economy, reducing FW and food loss while simultaneously generating profit and creating sustainable business models.

As contemporary food consumption structures evolve through digitalization, the need has emerged to understand how technological intermediaries shape waste. In seventh place, the article “Over-Ordering and Food Waste: The Use of Food Delivery Apps During a Pandemic” [88] examined the impact of food delivery apps on waste behavior during the COVID-19 pandemic. The study identified that delivery apps often facilitate over-ordering through intentional design mechanisms that promote larger quantities. Moreover, during the pandemic lockdown, when app use became critical for food access, this behavior intensified.

Parallel to studies on household contexts during crises, researchers began extending behavioral paradigms to out-of-home food consumption. In eighth place, the study “What Influences Consumer Food Waste Behavior in Restaurants? An Application of the Extended Theory of Planned Behavior” [33] applied an expanded version of TPB to examine 386 restaurant consumers in different Turkish cities. The results identified that in restaurant contexts, price awareness and taste preference emerged as the most significant predictors of waste. Consumers who prioritized taste and variety reported higher waste, often ordering multiple dishes to experiment with different flavors. This work is distinctive for offering the first robust analysis of waste in out-of-home contexts, utilizing rigorous behavioral frameworks to identify that the determinants of restaurant waste differ significantly from those in household contexts.

Recognizing the growing complexity of factors influencing FW, researchers developed broader integrative perspectives. Ranked number nine, the article “Towards a Multi-Level Framework of Household Food Waste and Consumer Behaviour: Untangling Spaghetti Soup” [28], represents a systematic meta-analysis of 118 prior studies on household FW and consumer behavior. Its title refers to Quested et al.’s [89] earlier characterization of the complexity of interactions among multiple factors influencing waste. The critical contribution of this work was the institutionalization of a multilevel perspective, recognizing FW as a complex interaction across individual-level factors (knowledge, skills, attitudes, personal norms), household-level contexts (family composition, subsistence dynamics, storage availability), and macro-level contexts (social environments, regulatory policies, recycling infrastructure, food prices, cultural norms). The systematic analysis revealed that 56% of primary studies relied exclusively on survey methodologies, with a European bias, and more importantly, that predictive factors in one context may be irrelevant in others.

This theoretical reorientation toward multilevel perspectives directed growing attention to geographically diverse and economically differentiated contexts. Finally, in tenth place, the study “Rural Household Food Waste Characteristics and Driving Factors in China” [22] examined 207 rural households across three provincial regions in China, combining direct waste collection, surveys, and interviews. Factor analysis identified village type, household size, income, age structure, health status, pet ownership, and dietary diversity as the main drivers. Particularly relevant, larger households wasted less per capita, suggesting economies of scale in food management. One reason this study has been widely cited is that it provided relevant data on a non-European context, often neglected, documenting that waste patterns in rural Chinese areas differ from urban regions and from high-consumption European/American contexts.

Beyond their citation metrics, these ten articles represent seminal theoretical contributions that have shaped the field’s intellectual trajectory. Stancu et al. [21] fundamentally reconceptualized the dual-route model of FW, viewing it as both intentional and unintentional, thereby bridging cognitive psychology with practice theories. This theoretical innovation influenced subsequent debates about the relative importance of conscious attitudes versus unconscious habits. Similarly, Jribi et al. [87] COVID-19 study provided empirical evidence for structural change theories, demonstrating how exogenous shocks can reconfigure household routines more rapidly than psychological interventions.

The theoretical evolution reflected in these citations reveals a field in transition: from individual psychological models [29,31] toward systemic and contextual approaches [64,88]. This shift mirrors broader theoretical debates in environmental psychology and sustainability science about the limits of individual agency versus structural determinants. The high citation counts for these articles reflect not only methodological rigor but also theoretical significance; each represents a conceptual advance that has opened new research avenues or challenged existing paradigms.

Notably, citation patterns correlate with theoretical turning points. The 2016 cluster (Stancu, Visschers) established empirical foundations, while the 2020–2023 cluster (Jribi, Aschemann-Witzel, Sharma) represents the field’s maturation toward more complex, multi-level theoretical frameworks. This progression suggests that the field’s most influential works are those that successfully integrate empirical findings with theoretical innovation, moving beyond descriptive accounts to offer explanatory frameworks with broader applicability.

#### Convergences and Divergences Among the Most Cited Articles

Despite addressing different contexts, methodologies, and periods, the ten articles reveal convergences that structure the field’s consensus. There is agreement on the main behavioral drivers: all studies identify inadequate food planning, over-purchasing, inefficient storage habits, and limited use of leftovers as central factors, suggesting that these represent structural characteristics of modern food consumption behavior. All studies also highlight the inadequacy of purely psychological or informational models—that is, simple awareness that waste is environmentally harmful does not motivate significant behavioral change. This conclusion implies that policies focused solely on education and awareness campaigns face fundamental limitations. There is recognition that financial motivations often outweigh environmental ones, meaning that even in European studies, where environmental concerns are comparatively high, saving money often predominates as the primary driver of waste reduction. This reasoning suggests that policymakers should frame waste reduction through both economic and environmental lenses. Multiple studies also demonstrate that contextual and structural factors often outweigh individual determinants, shaping waste as much as psychological choices.

Alongside these convergences, important divergences structure the field and indicate directions for future research. A central tension revolves around the role of perceived capacities versus actual competencies: some studies emphasize that individuals who perceive themselves as competent in food management tend to waste less, suggesting that self-efficacy plays a critical role. However, studies in rural and developing contexts [22] suggest that actual structural capacities outweigh self-reported perceptions. A second divergence relates to mechanisms of behavioral change. While behavioral studies suggest that skill training and reinforcement of social norms are effective pathways, works on circular economy and pandemic contexts suggest that structural changes in the physical and economic environment often drive more significant behavioral transformations than psychological interventions. A third emerging tension involves the presumed universality of determinants: while early studies suggested that behavioral factors identified in Western Europe generalized to other contexts, later culturally diverse studies revealed that lower-income contexts, different family structures, and limited storage infrastructure generate fundamentally different waste patterns. A fourth divergence relates to the measurement methodology. Studies using direct waste collection often find different amounts of waste compared to self-reported surveys, suggesting that social desirability bias generates systematic underreporting.

## 4. Discussions

This study performed a bibliometric review on the research field related to consumer behavior and FW. Our study characterized the research community and identified key research themes and emerging trends. Our study builds upon previous reviews and bibliometric studies on FW, which have examined this phenomenon from various perspectives. Kaman et al. [90] conducted a systematic literature review on FW in restaurants, identifying themes ranging from operational processes to best practices through thematic analysis. Zhang and Jian [91] visually analyzed student-related FW literature in the WoS core collection database. Jiao and Qiao [92] quantitatively assessed the global research progress on FW, highlighting core topics and emerging hotspots that have shifted from treatment processes to quantification, environmental impacts, consumer behaviors, and interventions. In recent years, restaurant FW and quantification studies have gained prominence. Baybars et al. [93] explored the evolution of FW research using bibliometric methods, whereas Carvalho et al. [94] proposed principles for an Integrated Sustainable Food Waste Reduction System, combining public policy, consumer behavior, and technological innovation. Their systematic review and bibliometric analysis identified critical gaps and synthesized knowledge to support actionable strategies aligned with sustainability goals.

Corrado and Sala [95] reviewed global and European studies on FW generation, comparing methodological approaches and their potential to inform European policies. Amicarelli and Bux [96] examined FW measurement methodologies, identifying global approaches, limitations, and opportunities. Chen et al. [39] conducted a bibliometric study analyzing the chronological distribution, countries, institutions, source titles, subject categories, and keywords, revealing a rapid growth in FW research over the past 18 years. Elgahary et al. [97] reviewed global approaches to converting FW into usable products and green energy. Our study fills the existing gaps in the literature by explicitly focusing on FW and consumer behavior.

Our study builds upon previous reviews and bibliometric studies on FW by examining the interplay between consumer behavior and FW. Based on the reported findings, our study also aims to identify some directions for future research, addressing RQ4. Our study observed that the systematic analysis of high-impact literature on consumer-level FW reveals a rapidly expanding research field, but one marked by significant theoretical, methodological, and geographical concentrations. Despite advances in identifying the determinants of FW, the phenomenon—often described as a “spaghetti soup” of complex interactions [89]—still requires a more integrated and multifaceted approach.

Theoretical challenges highlight a gap between intention and routine. In this regard, the TPB has proven to be a fundamental framework, but its predictive efficacy is limited when waste behavior is not entirely under intentional control. Studies have shown that routines related to shopping and leftover reuse, along with Perceived Behavioral Control, are stronger predictors than mere intention [33,98,99,100].

Methodologically, the dominance of self-report surveys represents a substantial limitation, since it primarily relies on participants’ memory [101]. Hence, it is essential to acknowledge some of its main limitations, including the tendency for people to underestimate behaviors related to adverse environmental impacts or socially rejected actions, inaccuracies due to external influences that can generate fluctuations [102], and underestimation of FW levels [103,104]. Despite these limitations, it has been one of the most used methods in FW research [105].

Some actions can be recommended to overcome these limitations, such as direct measurements or triangulation of methods [106], use of mixed-methods to achieve triangulation and completeness [107], FW audits, and the usage of digital technologies. Parizeau et al. [108] argue that household survey data should be used in conjunction with the direct observation of FW via FW audits. FW audits create opportunities for monetary savings and identify areas and initiatives for further reduction of FW. However, they are usually labor-intensive and require understanding the scope of the problem, setting FW reduction goals, monitoring progress, assessing interventions, and coordinating staff [109]. Cook et al. [110] state that the burdens of FW measurement will decrease with the introduction of digital technologies. They can support FW reduction and management, primarily in the production and distribution phases, through the use of smart bins, waste-sorting robots, and predictive models [111]. Smart bins utilize sensors, data analytics, and automation, enabling real-time monitoring of FW levels, route optimization, and promoting efficient refuse disposal strategies [112]. At the consumer level, AI-powered apps and dynamic pricing systems have presented potential benefits [113]. Barbosa and Gomes [114] stated that data science and sentiment analysis could be used to analyze FW online discussions. Anyway, researchers claim that digital solutions need to be tailored to specific consumer segments (income levels, country, among other characteristics) [113]. Additionally, challenges related to data limitations, costs, and integration need to be addressed [111].

Geographically, most evidence originates from high-income countries, with a notable concentration in European studies [97,115,116]. Despite this fact, some studies in our sample have been carried out in emerging markets. In developed nations, most FW happens at the consumer/retail level due to overbuying and cosmetic standards, while in developing countries, waste occurs earlier in the supply chain (harvest, storage, transport) due to poor infrastructure, technology, and processing [117,118]. Although some developing countries are, on one hand, significant producers and exporters of agricultural products, and on the other hand, countries with malnutrition and food scarcity, they are also notable producers of FW and offer unique contexts for investigation [119]. Probably due to a perception of abundance of food, FW is perceived as relatively high in countries like Uruguay [72]. Culture is also a determinant of FW in countries like Brazil [120] and Iran [121], where food volume and diversity are associated with hospitality. There is an urgent need to validate models in developing countries, where drivers of FW may differ significantly.

Based on these findings, the field would benefit from investigating the role of moral norms, specifically responsibility denial, to understand when aversion to waste is activated or ignored. In addition, the development of systemic solutions, such as the concept of upcycled foods, which divert valuable ingredients from waste, offers an opportunity to study consumer acceptance and the effectiveness of communication strategies that emphasize resource efficiency [122]. Future interventions should move beyond mere awareness (which has limited impact when isolated) and focus on improving perceived skills and providing practical tools for routines.

Based on the findings of our study, we propose several future research directions related to the identified research streams. Table 5 presents a map of research gaps and recommendations to guide future investigations. The References column includes citations to studies that ground our research proposals.

Table 5 presents some research gaps and potential paths for future studies, organized into four distinct research streams. The first stream is Systemic approaches. Current research on household FW remains predominantly focused on individual-level psychological factors, creating a significant theoretical gap in understanding the multi-level systemic interactions that shape waste generation patterns. As Boulet et al. [28] compellingly argue, this reductionist perspective overlooks the complex interplay between micro-level behaviors, meso-level institutional contexts, and macro-level structural factors. This limitation is echoed by Roodhuyzen et al. [20], who critique the field’s fragmentation and call for more integrated analytical frameworks. To bridge this gap, researchers must adopt ecological models of behavior, which recognize the nested environmental influences on individual actions. The work of Heikkilä et al. [128] demonstrates how organizational factors in food service settings interact with consumer behaviors, while Engström and Carlsson-Kanyama [127] highlight the importance of considering entire food life cycles. Furthermore, approaches such as those employed by Beretta and Hellweg [126], which analyze environmental impacts across product lifecycles, provide essential methodological tools for systemic analysis. By integrating these multi-level perspectives, researchers can develop more comprehensive interventions that address leverage points throughout the food system rather than focusing exclusively on individual behavior change.

The second proposed research stream is related to socio-economic segmentation. The geographical and socio-economic concentration of food waste research in developed Western countries represents a critical knowledge gap, limiting our understanding of how waste patterns vary across different economic, cultural, and developmental contexts. As Li et al. [22] demonstrate through their study of rural Chinese households, waste generation in developing economies follows fundamentally different patterns, with significantly lower per capita waste compared to high-income countries. This contextual variability is further evidenced by Koivupuro et al. [129], who identified household size and income as significant predictors of waste in Finland, and Parizeau et al. [130], who found positive correlations between food expenditures and waste generation in Canada. To address this segmentation gap, research must expand systematically into emerging economies and diverse socio-economic settings. Research must incorporate cultural anthropology approaches to understand local food practices and norms, as demonstrated by Filimonau et al. [70] in their examination of food sharing traditions. The work of Coskun and Yetkin Özbük [33] further illustrates how cultural factors mediate the effectiveness of waste reduction strategies. Future investigations should particularly focus on how rapid urbanization and income growth in transitional economies affect waste patterns, while also examining the specific barriers faced by rural households in implementing reduction strategies. This contextualized understanding is essential for developing culturally appropriate interventions that respect local food systems and economic realities.

Policy Experimentation is the subject of the third proposed research stream. Despite growing policy attention to reducing FW, a significant implementation gap persists between theoretical solutions and empirically validated interventions, with overreliance on informational campaigns that demonstrate limited effectiveness. As Schanes et al. [132] systematically review, most existing policies lack robust evidence of impact, while Jribi et al. [87] specifically document the inefficiency of information-only campaigns during the COVID-19 pandemic. This evidence gap is compounded by perceived legal barriers to food donation and redistribution, as well as insufficient testing of behavioral interventions in real-world settings. To advance policy effectiveness, rigorous experimentation with diverse intervention types is urgently needed. Randomized controlled trials (RCTs) of behavioral “nudges,” such as those examining portion size adjustments and feedback mechanisms, provide essential evidence for policy design. Simultaneously, research must address structural barriers through legal analysis and stakeholder engagement. The implementation of practical measures to achieve SDG 12.3, which aims to halve per capita food waste by 2030, requires systematic monitoring and evaluation frameworks, such as those proposed by Beretta and Hellweg [126] for tracking progress toward reduction targets. Furthermore, natural experiments leveraging policy changes, combined with cost–benefit analyses of different intervention types as advocated by Reynolds et al. [131], can identify the most efficient allocation of resources. By grounding policy development in empirical evidence and addressing both behavioral and structural determinants, researchers can contribute to more effective, scalable solutions that account for the diverse contexts in which food waste occurs.

The final stream is related to methodological innovations related to FW reduction and management. The persistent reliance on self-reported surveys and questionnaires constitutes a fundamental methodological gap in food waste research, leading to systematic underestimation and social desirability bias that compromise data integrity and the effectiveness of policies. As demonstrated by Visschers et al. [24] and Giordano et al. [125], self-report instruments are inherently unreliable for quantifying actual waste levels, with respondents consistently underreporting their food disposal behaviors. This methodological limitation is further corroborated by Neff et al. [31], who documented substantial discrepancies between reported awareness and actual waste generation among U.S. consumers. To address these measurement challenges, the field must transition toward more objective, technology-enhanced methodologies. Direct weighing studies, as implemented by Betz et al. [123] in Swiss households, provide more accurate baseline data. The integration of smart bin technologies with weight sensors, combined with longitudinal observational methods as employed by Silvennoinen et al. [124], offers promising avenues for real-time monitoring without respondent interference. Furthermore, mixed-method approaches that triangulate survey data with direct measurement, as advocated by Coskun and Yetkin Özbük [33].

## 5. Conclusions

The results of this bibliometric analysis allow us to conclude that the field of studies on FW associated with consumer behavior has undergone, over the past decade, a process of accelerated expansion, consolidating itself as a mature, plural, and methodologically diverse area. Based on the analysis of 229 articles from the WoS database, it was possible to identify not only the temporal evolution of scientific production but also the formation of collaboration networks, central and emerging themes, and conceptual and methodological gaps, as well as opportunities for advancement for researchers, managers, and policymakers. As a major conclusion, it is emphasized that the relationship between FW and consumer behavior has become a pivotal axis of contemporary debates on sustainability, encompassing psychosocial determinants, household routines, and systemic and contextual factors that shape everyday food practices.

It was observed that much of the literature focuses on psychosocial models—especially the TPB—and self-reported measures, proposing that attitudes, norms, and intentions are central elements in explaining waste behavior. However, the findings of this study show that routinized practices, cognitive biases, structural limitations, and situational elements have increasingly been incorporated into the debate, suggesting that FW cannot be understood solely through the lens of individual intentions. Consumer behavior thus emerges as the result of a complex interaction among micro-level factors (motivations, values, perceptions), meso-level factors (family dynamics, life cycles, household composition), and macro-level factors (economic context, public policies, urban and retail infrastructure). This complexity underscores the importance of adopting integrated, multi-level, and methodologically robust approaches to enhance understanding of the phenomenon.

From an academic perspective, this study offers substantial contributions by organizing the state of the art, identifying influential authors, mapping the research community, and revealing methodological and theoretical patterns that structure the field. For researchers, the results assist in understanding the main intellectual currents, the themes that have guided investigations, and the gaps that remain open—among them, the need for studies that articulate practices and contexts, deepen research in the Global South, and expand methodologies beyond self-reported surveys. In addition, the mapped evidence may encourage international collaborations, diversify perspectives, and enhance comparative investigations across diverse socioeconomic contexts.

For policymakers and managers, the findings of this research offer relevant insights, demonstrating that consumer behavior is sensitive to interventions that combine information, infrastructure, economic incentives, regulation, and the design of food systems. The finding that financial factors and shopping routines have a significant impact on waste generation underscores the need for policies that facilitate meal planning, reduce the use of inadequate packaging, enhance labeling, and promote campaigns aimed at reducing leftovers and promoting a proper understanding of expiration dates. In addition, the identification of emerging themes, such as upcycled foods and the impacts of food delivery apps, opens space for innovative policies that integrate circular economy principles, food education, and regulation of digital commerce. Our study suggests that public policies should promote FW interventions that are multi-level and context-sensitive, integrating economic and behavioral insights.

Finally, it is important to recognize some limitations of this study. The bibliometric analysis depends directly on the selected database, and despite the comprehensiveness of WoS, relevant studies present in other databases may not have been included. To strengthen future analyses, data could be supplemented with records from additional repositories, such as Scopus, thereby broadening coverage while maintaining quality. Moreover, the interpretation of results is conditioned by the information available in the article metadata, which limits deeper qualitative analyses of content and methodology. Finally, to ensure the retrieval of closely related articles, the search was restricted to articles with terms related to FW and consumer behavior in their titles. The use of different search terms may allow for the identification of other relevant articles. Future research is encouraged to integrate automated textual analyses, comparisons across different databases, and complementary studies that directly examine consumer practices and discourses in diverse cultural contexts.

In summary, this study contributes to the understanding of the dynamics that structure FW in the realm of consumer behavior, offering a comprehensive overview and strategic guidance for scientific advancement and for transformative actions capable of addressing this global challenge.

## Figures and Tables

**Table 1:** Main researchers in terms of number of published articles.

Reference	Type of Study	Search Period	Searched Databases	Objectives	Techniques/Main Findings
Gál et al. [1]	Bibliometric analysis	2019–2024	WoS and Scopus	Identification of gaps for future research on FW and sustainable FW management.	Identification of highly cited documents, research areas, and keyword co-occurrence patterns.
Zhang et al. [41]	Bibliometric analysis	1991–2015	WoS	Identification of FW research activities and tendencies.	Main research themes, top publishing countries, cluster analysis
Widayat et al. [9]	Systematic literature review	2015 and 2023	Scopus and PubMed	Analysis of consumer behavior related to food waste within the fields of management, business, economics, and social sciences.	Thematic analysis.
Moraes et al. [42]	Systematic Literature Review (SLR)	Up to 2020	Science Direct, Scopus, Wiley, and WoS.	Identification of FW prevention and minimization methods.	Classification of the prevention and minimization methods and suggestions of future research, proposal of a research agenda to indicate trends
Batool et al. [43]	Bibliometric analysis.	2005–2023	Scopus	Analysis of FW management methods based on lifecycle assessment (LCA) techniques.	Analysis of relevant data trends and patterns.
Wang et al. [44]	Bibliometric analysis	Up to 2023	WoS	Assessment of the environmental impact of FW reduction on agri-food systems.	Proposal of a conceptual framework and identification of research themes.
Chen et al. [39]	Bibliometric analysis	1997–2014.	WoS	Identification of the state of the art of research on FW	Characterization of the research community and source titles and author keywords.
Vittuari et al. [45]	Systematic literature review	2010–2021	WoS	To provide a systematic overview of FW drivers and levers.	Proposal of indications of potential consumer FW reduction interventions.

**Table 2:** Top publishing authors.

Ranking	Researcher	Affiliation	Publication Year									Number of Published Articles	% of Published Articles *
			2016	2017	2018	2019	2020	2021	2022	2023	2024		
1	Aschemann-Witzel, Jessica	Aarhus University—Denmark		2	4	1	1	1	1	2		12	5.2
2	Ares, Gaston	Aarhus University—Denmark			3	1	1		1	1		7	3.1
3	Gimenez, Ana	Universidad de la Republica—Uruguay			2	1	1		1	1		6	2.6
4	Vittuari, Matteo	University of Bologna—Italy	1			1	1	1	1	1		6	2.6
5	Filimonau, Viachaslau	University of Surrey—United Kingdom				1	1		1	2		5	2.2
6	Falasconi, Luca	University of Bologna—Italy	1			1	1	1				4	1.7
7	Kasza, Gyula	University of Veterinary Medicine Budapest—Hungary			1		1	1	1			4	1.7
8	Pearson, David	Central Queensland University—Australia						1	1	1	1	4	1.7
9	Segre, Andrea	University of Bologna—Italy	1			1	1	1				4	1.7
10	Setti, Marco	University of Bologna—Italy	1			1	1		1			4	1.7
	Total											56	24.5

* Note: Sample of 229 articles; Source: based on WoS data (2025).

**Table 3:** Most impactful researchers.

Ranking	Researcher	h-Index	g-Index	m-Index	Publication Year of the First Article
1	Aschemann-Witzel, Jessica	12	12	1.33	2017
2	Ares, Gaston	6	7	0.75	2018
3	Gimenez, Ana	6	6	0.75	2018
4	Vittuari, Matteo	6	6	0.60	2016
5	Filimonau, Vachaslau	5	5	0.71	2019

Source: Adapted from Bibliometrix, based on WoS data (2025).

**Table 4:** Most cited articles.

Ranking	Article Title	Authors	Publication Year	Total Number of Citations	Average Number of Citations per Year
1	Determinants of consumer food waste behavior: Two routes to food waste	Stancu, Violeta; Haugaard, Pernille; Lahteenmaki, Liisa	2016	738	73.8
2	Sorting out food waste behavior: A survey on the motivators and barriers of self-reported amounts of food waste in households	Visschers, Vivianne H. M.; Wickli, Nadine; Siegrist, Michael	2016	576	57.6
3	COVID-19 virus outbreak lockdown: What impacts on household food wastage?	Jribi, Sarra; Ben Ismail, Hanen; Doggui, Darine; Debbabi, Hajer	2020	322	53.7
4	Avoiding food waste by Romanian consumers: The importance of planning and shopping routines	Stefan, Violeta; Van Herpen, Erica; Tudoran, Ana Alina; Lahteenmaki, Liisa	2013	540	41.5
5	Wasted Food: US Consumers’ Reported Awareness, Attitudes, and Behaviors	Neff, Roni A.; Spiker, Marie L.; Truant, Patricia L.	2015	319	29.0
6	Defining upcycled food: The dual role of upcycling in reducing food loss and waste	Aschemann-Witzel, Jessica; Asioli, Daniele; Banovic, Marija; Perito, Maria Angela; Peschel, Anne Odile; Stancu, Violeta	2023	82	27.3
7	Over-ordering and food waste: The use of food delivery apps during a pandemic	Sharma, Rajat; Dhir, Amandeep; Talwar, Shalini; Kaur, Puneet	2021	130	26.0
8	What influences consumer food waste behavior in restaurants? An application of the extended theory of planned behavior	Coskun, Aysen; Yetkin Ozbuk, Raife Meltem	2020	146	24.3
9	Towards a multi-level framework of household food waste and consumer behavior: Untangling spaghetti soup	Boulet, Mark; Hoek, Annet C.; Raven, Rob	2021	119	23.8
10	Rural household food waste characteristics and driving factors in China	Li, Yunyun; Wang, Ling-en; Liu, Gang; Cheng, Shengkui	2021	114	22.8

Source: Based on Web of Science data.

**Table 5:** Research Gaps and Recommendations for Future Studies.

Research Stream	Current Gap	Concrete Research Questions	Concrete Research Methods/Approaches	Expected Theoretical Contribution	Practical Implications	References
Systemic Approaches	Focus on individual-level factors overlooks multi-level complexity and systemic interactions	“How do municipal waste collection policies interact with household storage practices to influence food waste?” “What is the relative importance of packaging design vs. consumer education in reducing waste of perishable foods?” “How do food delivery platforms reshape traditional household food management routines?”	1. Hierarchical Linear Modeling (HLM) to analyze multi-level interactions 2. Agent-based modeling of household decision-making 3. System dynamics modeling of food supply chains 4. Network analysis of food flow within communities	Integrated theoretical models that bridge individual psychology with structural determinants	Holistic policy interventions that address multiple leverage points simultaneously	[24,31,33,123,124,125]
Socio-economic Segmentation	Geographic concentration in developed countries; lack of research in diverse socio-economic contexts	“Do low-income households in developing countries waste food for different reasons than high-income households in developed countries?” “How do cultural norms around food sharing and hospitality affect waste patterns in collectivist vs. individualist societies?” “What specific barriers prevent rural households from implementing food waste reduction strategies?”	1. Comparative studies between developed and developing countries 2. Rural-urban comparisons using standardized protocols 3. Income-stratified analysis of waste drivers 4. Cultural anthropology approaches to understand local food practices	Context-sensitive theories that account for economic, cultural, and geographic variability	Tailored interventions for specific population segments; culturally appropriate communication strategies	[20,28,126,127,128]
Policy Experimentation	Disconnection between policy solutions and behavioral determinants; limited evidence on intervention effectiveness	“Which combination of nudges (information, feedback, incentives) is most cost-effective in reducing restaurant food waste?” “How do deposit-refund systems for food containers compare to awareness campaigns in reducing packaging waste?” “What are the unintended consequences of food waste taxation on different socio-economic groups?”	1. Randomized controlled trials (RCTs) of different policy instruments 2. Natural experiments leveraging policy changes 3. Cost–benefit analysis of waste reduction programs 4. Stakeholder analysis of policy implementation barriers	Evidence-based policy frameworks with clear causal mechanisms	Scalable policy solutions with proven effectiveness; optimized resource allocation for maximum impact	[22,32,33,129,130]
Methodological Innovation	Strong dependence on self-report surveys leading to social desirability bias and data inaccuracy	“Does the use of smart bins with weight sensors reduce social desirability bias compared to self-reported diaries?” “How do measurement methods (weighing vs. estimation) affect the identified determinants of food waste?” “Can machine learning algorithms accurately predict food waste from household purchasing patterns?”	1. Direct weighing studies in diverse household settings 2. Smart bin technology with sensors for real-time tracking 3. Mixed-methods approaches combining surveys with observational data 4. Longitudinal diary studies with photo documentation	Development of validated measurement frameworks that account for contextual factors and behavioral complexity	More accurate baseline data for policy design; improved evaluation of intervention effectiveness	[87,126,131,132]

## Data Availability

The original contributions presented in this study are included in the article. Further inquiries can be directed to the corresponding author.

## Abbreviations

The following abbreviations are used in this manuscript:

FW	Food waste
FAO	Food and Agriculture Organization
SDGs	Sustainable Development Goals
TPB	Theory of Planned Behavior
WoS	Web of Science
USA	United States of America
HLM	Hierarchical Linear Modeling
FDA	Food Delivery Apps
